# Supplementary material for: Intravesicular Genomic DNA Enriched by Size Exclusion Chromatography Can Enhance Lung Cancer Oncogene Mutation Detection Sensitivity
Source: Int J Mol Sci. 2022 Dec 16;23(24):16052. doi: 10.3390/ijms232416052 (PMC9785009; doi:10.3390/ijms232416052)
Supplement: Supplementary file 1 [file ijms-23-16052-s001.zip › Supplementary Figure S1.pdf]

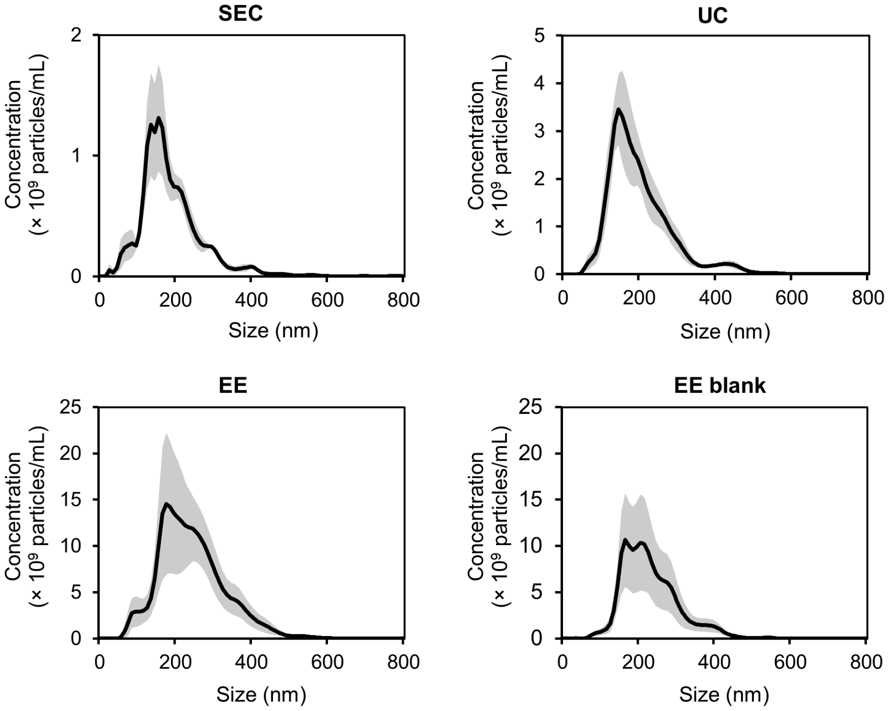

Supplementary Figure S1. Size distribution profiles of H1975 sEV fractions and blank controls by scatter-based NTA. For each method, the mean size distribution profile of the sEV fraction and blank control (solid line) is shown with the SEM (shaded area, N = 3, n = 3). For SEC and UC, the number of particles present in the blank was too low to perform an accurate measurement (data not shown). Profiles represent the undiluted sEV sample concentrations separated from 90 mL conditioned media (SEC and UC) or 45 mL conditioned media (EE).
