# Supplementary material for: Intravesicular Genomic DNA Enriched by Size Exclusion Chromatography Can Enhance Lung Cancer Oncogene Mutation Detection Sensitivity
Source: Int J Mol Sci. 2022 Dec 16;23(24):16052. doi: 10.3390/ijms232416052 (PMC9785009; doi:10.3390/ijms232416052)
Supplement: Supplementary file 1 [file ijms-23-16052-s001.zip › Supplementary Figure S2.pdf]

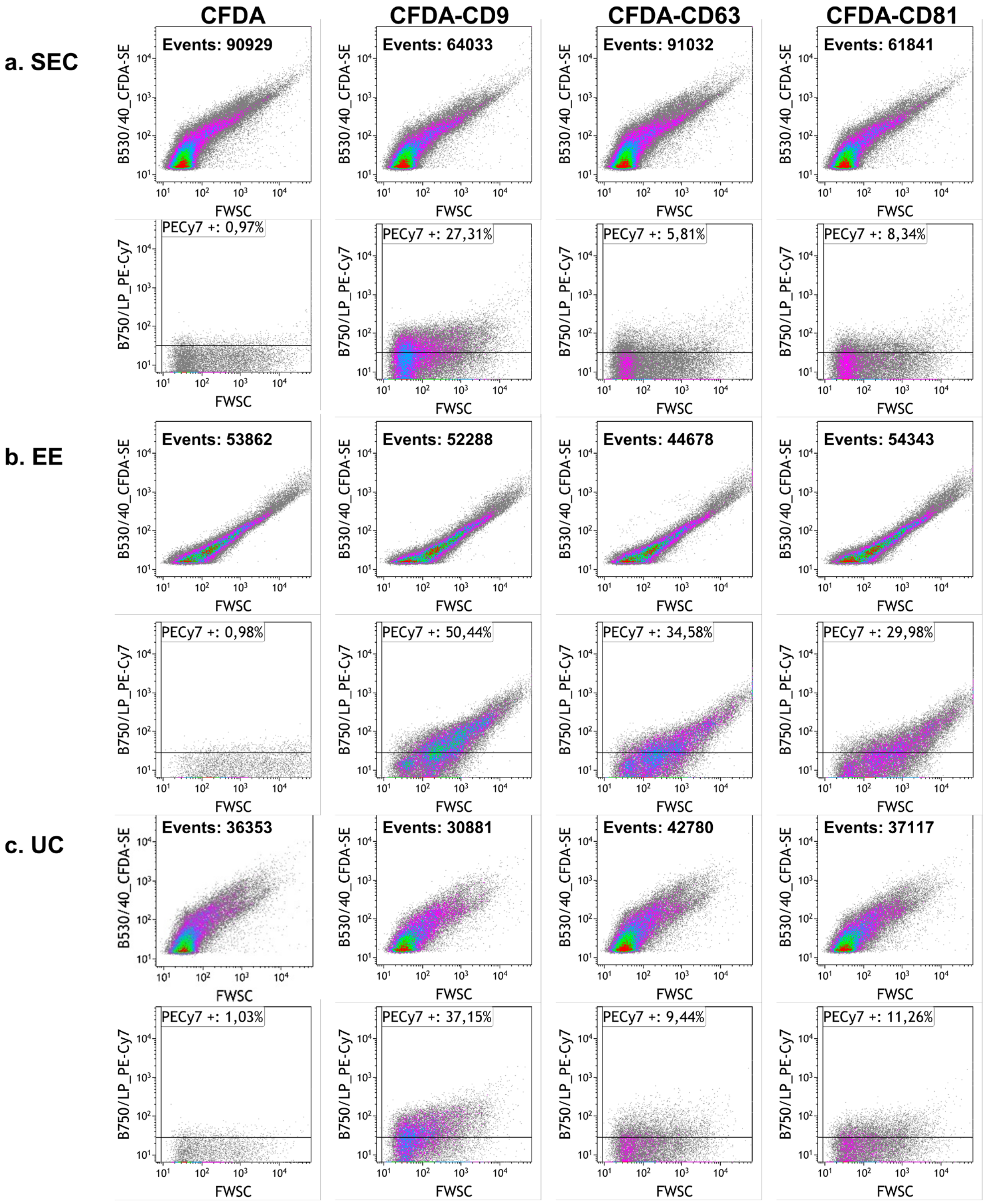

Supplementary Figure S2. Flow cytometry of H1975 sEV fractions obtained by SEC (a), EE (b) and UC (c). Samples were CFDA-SE single (CFDA) or double stained with anti-human CD9-PECy7 (CFDA-CD9), CD63-PECy7 (CFDA-CD63) or CD81-PECy7 (CFDA-CD81) fluorescent antibodies. Fluorescence intensities (y-axis) of CFDA-SE and PECY7 are plotted against forward scatter (FWSC, x-axis). EVs were measured by fluorescence triggering on CFDA-SE with a threshold of 0.30 set at the FL-1 fluorescent channel (B530/40) and gates were set on the FL-2 fluorescent channel (B750/LP) using CFDA-SE single-stained samples, so that the percentage of positive events was ≈1%. Representative dot plots out of 2 independent experiments (n = 3) are shown.
