# Supplementary material for: Intravesicular Genomic DNA Enriched by Size Exclusion Chromatography Can Enhance Lung Cancer Oncogene Mutation Detection Sensitivity
Source: Int J Mol Sci. 2022 Dec 16;23(24):16052. doi: 10.3390/ijms232416052 (PMC9785009; doi:10.3390/ijms232416052)
Supplement: Supplementary file 1 [file ijms-23-16052-s001.zip › Supplementary Figure S3.pdf]

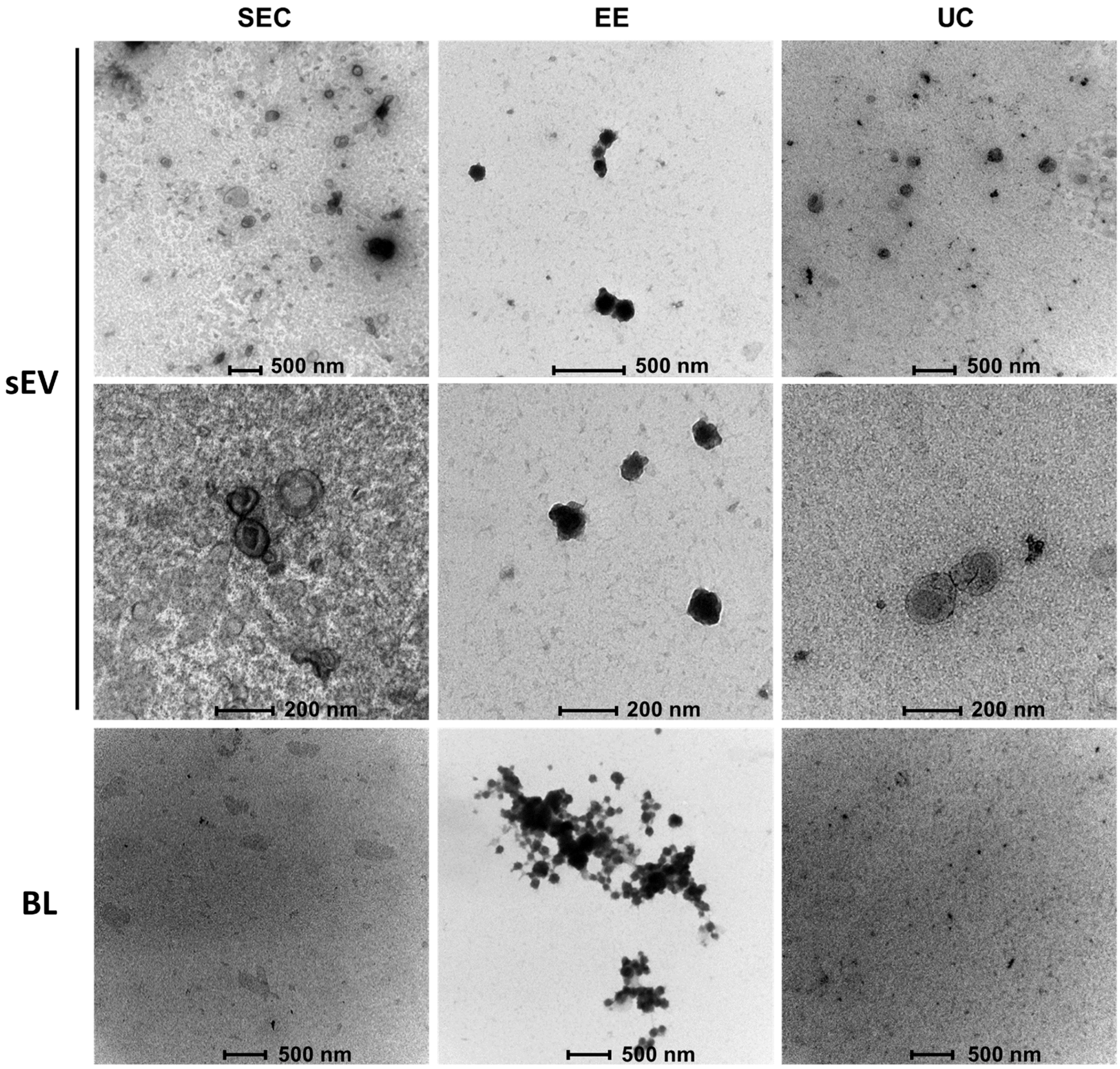

Supplementary Figure S3. Characterization of H1975 sEV fractions and blank controls by TEM. Images of sEV samples (sEV) are represented with a scale bar of 500 nm (top) and 200 nm (middle), and blank controls (BL) with a scale bar of 500 nm (bottom).
