# Supplementary material for: Intravesicular Genomic DNA Enriched by Size Exclusion Chromatography Can Enhance Lung Cancer Oncogene Mutation Detection Sensitivity
Source: Int J Mol Sci. 2022 Dec 16;23(24):16052. doi: 10.3390/ijms232416052 (PMC9785009; doi:10.3390/ijms232416052)
Supplement: Supplementary file 1 [file ijms-23-16052-s001.zip › Supplementary Figure S4.pdf]

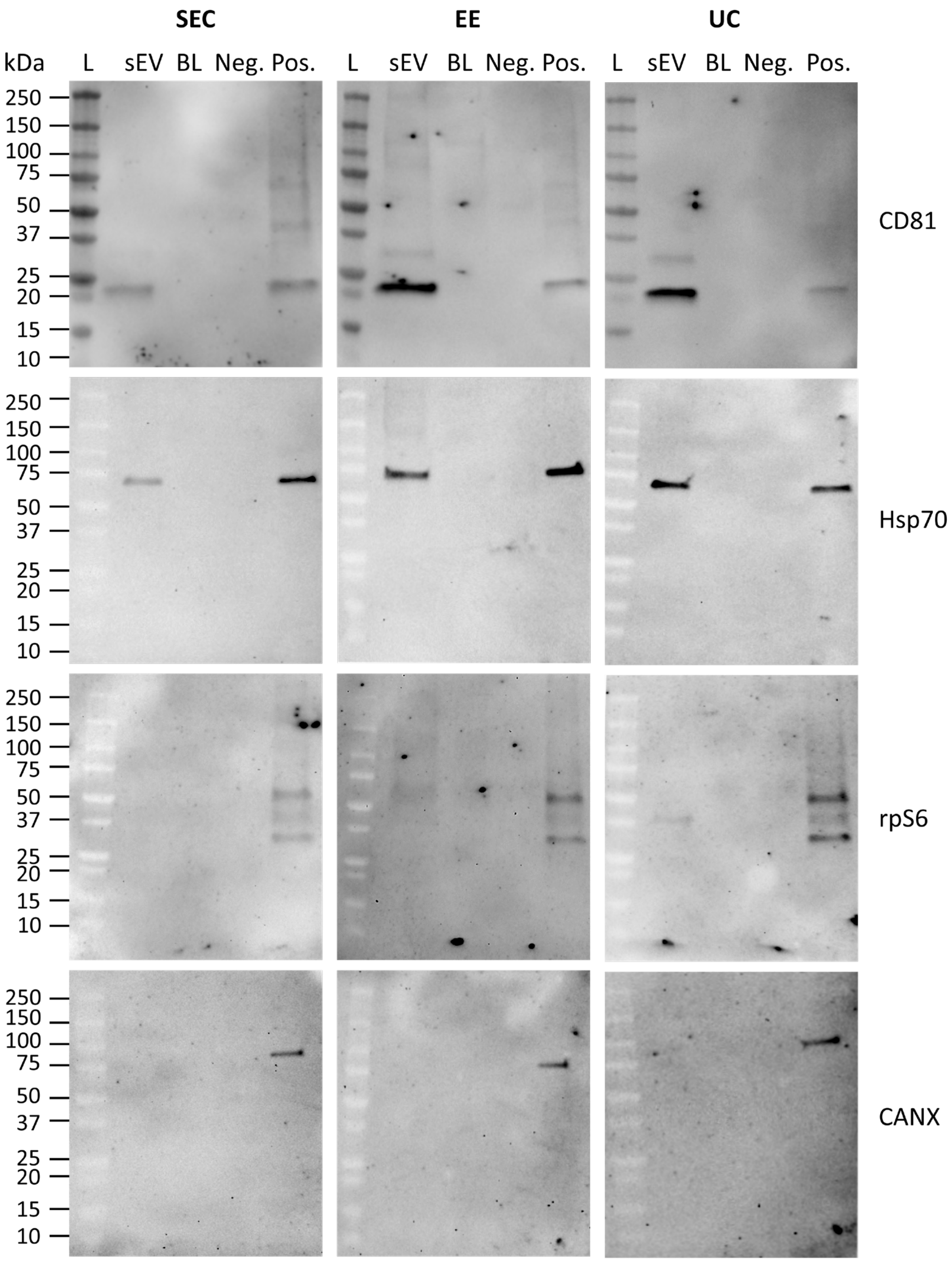

Supplementary Figure S4. Full images of the Western blot analyses in H1975 sEV samples. Proteins considered as EV-specific markers (CD81, MW = 24-26 kDa and Hsp70, MW = 70 kDa) and non-sEV markers (rpS6, MW = 34 kDa and CANX, MW = 90 kDa) were analyzed. Unconditioned RPMI-1640 + 2% EV-depleted FBS was used as a negative control (Neg.) and H1975 whole cell lysate as a positive control (Pos.). Equal volumes of the sEV samples, blank controls, positive and negative controls were used for analysis. L = ladder (kDa), sEV = sEV sample, BL = blank control.
