# Supplementary material for: Intravesicular Genomic DNA Enriched by Size Exclusion Chromatography Can Enhance Lung Cancer Oncogene Mutation Detection Sensitivity
Source: Int J Mol Sci. 2022 Dec 16;23(24):16052. doi: 10.3390/ijms232416052 (PMC9785009; doi:10.3390/ijms232416052)
Supplement: Supplementary file 1 [file ijms-23-16052-s001.zip › Supplementary Figure S5.pdf]

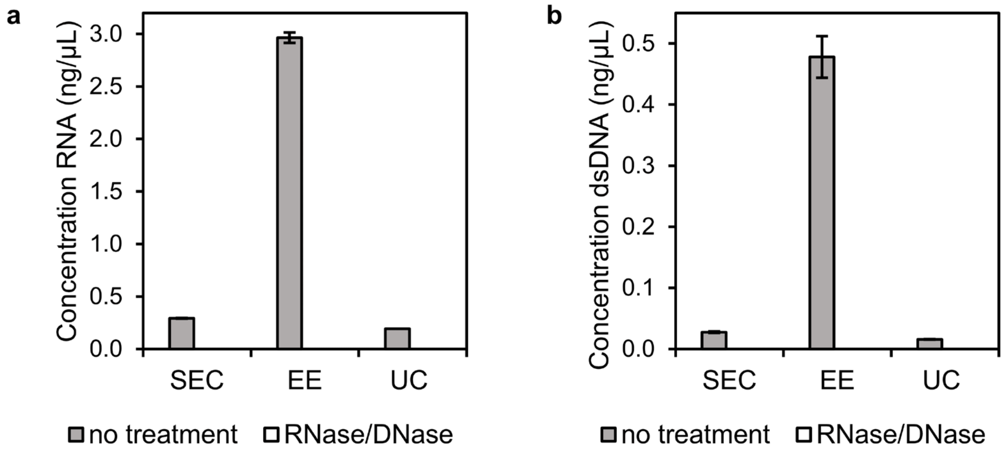

Supplementary Figure S5. Validation of the RNase/DNase treatment. Concentration measured using Qubit assays: (**a**) HS RNA and (**b**) HS dsDNA for samples obtained by the different workflows SEC, EE and UC. Total sEV-RNA/DNA extraction was performed on sEV samples obtained by the different methods without and with additional nuclease treatment before sEV lysis as described in the materials and methods section. Data is represented as mean ± SD of 2 separate sEV isolations.
