# Supplementary material for: Intravesicular Genomic DNA Enriched by Size Exclusion Chromatography Can Enhance Lung Cancer Oncogene Mutation Detection Sensitivity
Source: Int J Mol Sci. 2022 Dec 16;23(24):16052. doi: 10.3390/ijms232416052 (PMC9785009; doi:10.3390/ijms232416052)
Supplement: Supplementary file 1 [file ijms-23-16052-s001.zip › Supplementary Figure S6.pdf]

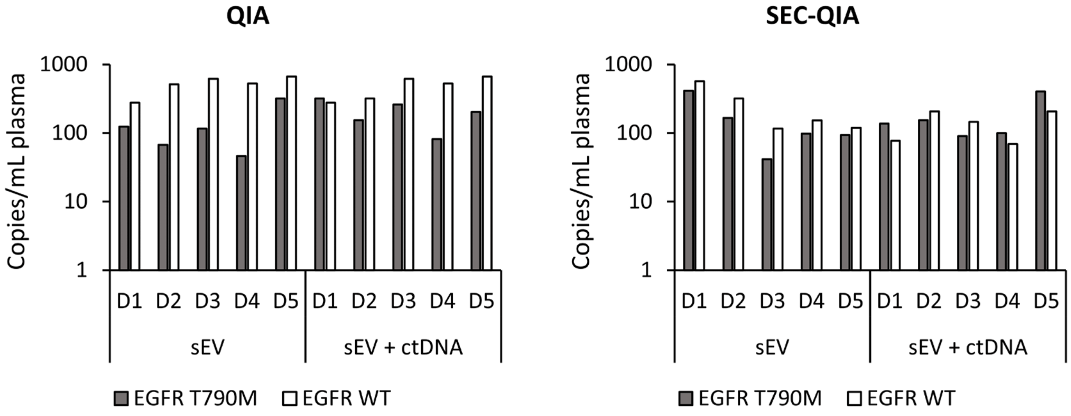

Supplementary Figure S6. *EGFR* copies per mL plasma of PPP-derived samples obtained by ddPCR. H1975-derived sEV-spiked plasma samples were prepared with 1 × 10^8 sEVs/mL plasma (sEV), or sEVs in combination with 300 copies short *EGFR* T790M DNA fragments (sEV + ctDNA), and processed either by the standard cell-free DNA extraction workflow (QIA) or by first separating the sEVs using SEC (SEC-QIA). All data of 5 individual donors is shown and represented as mean ± SD (N = 1, n = 2).
