# Supplementary material for: Intravesicular Genomic DNA Enriched by Size Exclusion Chromatography Can Enhance Lung Cancer Oncogene Mutation Detection Sensitivity
Source: Int J Mol Sci. 2022 Dec 16;23(24):16052. doi: 10.3390/ijms232416052 (PMC9785009; doi:10.3390/ijms232416052)
Supplement: Supplementary file 1 [file ijms-23-16052-s001.zip › Van Hoof_Supplementary Materials and Methods.pdf]

# Supplementary Materials and Methods

# Intravesicular genomic DNA enriched by size exclusion chromatography can enhance lung cancer oncogene mutation detection sensitivity

Rebekka Van Hoof[a,b,c], Sarah Deville[a,d], Karen Hollanders[a], Pascale Berckmans[a], Patrick Wagner[b], Jef Hooyberghs[c,e], Inge Nelissen[a*]

[a] *Health Unit, Flemish Institute for Technological Research (VITO), 2400 Mol, Belgium;*

[b] *Laboratory for Soft Matter and Biophysics, KU Leuven, 3000 Leuven, Belgium;*

[c] *Theoretical Physics, Hasselt University, 3590 Diepenbeek, Belgium;*

[d] *Biomedical Research Institute, Hasselt University, 3590 Diepenbeek, Belgium;*

[e] *Data Science Hub, Flemish Institute for Technological Research (VITO), 2400 Mol, Belgium*

** Corresponding author: Dr. Inge Nelissen, Boeretang 200, 2400 Mol, Belgium, +3214335211, inge.nelissen@vito.be*

*Note: Sarah Deville has moved to a different institution since the work was conducted. Current affiliations: Laboratory of Experimental Cancer Research, Department of Human Structure and Repair, Ghent University, 9000 Ghent, Belgium; Cancer Research Institute Ghent, 9000 Ghent, Belgium*

**General methodologies**

Throughout the study, PBS without $Ca^{2+}$ or $Mg^{2+}$ filtered over a 0.1 µm cut-off filter was used. All culture media were filtered over a 0.22 µm filter after addition of 2% EV-depleted fetal bovine serum (FBS, Gibco, #A2720801).

Protein LoBind microcentrifuge tubes were used after sEV separation. Aliquots were taken to avoid freeze-thaw cycles. Small EV-RNA/DNA samples were kept in DNA LoBind microcentrifuge tubes. All samples were stored at -80°C.

**ExoEasy membrane affinity chromatography**

Binding buffer (45 mL) was added to the conditioned cell media or blank controls and mixed gently. The mixture was passed through the exoEasy spin column by centrifugation at room temperature, 500 rcf for 1 minute in a swinging bucket Rotanta 460 R centrifuge with maximal brake. Ten mL of washing buffer was added to the column and spun down at room temperature, 4500 rcf for 5 minutes. The spin column was transferred to a new collection tube and 500 µL of exoEasy elution buffer was added, incubated for 1 minute at room temperature and centrifuged at 500 rcf for 5 minutes. The eluate was placed back on the column-membrane, incubated for 1 minute and centrifuged at 4500 rcf for 5 minutes.

**Characterization of fractions obtained by the different sEV separation methods**

*Protein concentration determination*

The DC Protein Assay (Bio-Rad Laboratories, #5000112) and the Micro BCA™ Protein Assay Reagent Kit (Thermo Fisher Scientific, #23235) were used to determine the protein concentration. These kits have a linear working range for bovine serum albumin of 200 µg/mL to 1500 µg/mL and 0.5 µg/mL to 20 µg/mL, respectively. A standard curve of Pierce™ Bovine Serum Albumin Standard Pre-Diluted Set (Thermo Fisher Scientific, #23208) of 125 - 250 - 500 - 750 - 1000 - 1500 - 2000 µg/mL for the DC Protein Assay, and a dilution series of the 2000 µg/mL solution to 0.5 - 1 - 2.5 - 5 - 10 - 20 - 40 - 200 µg/mL in PBS for the Micro BCA Protein Assay Reagent Kit, together with PBS as blank were used to calculate the protein concentration. SEC samples were measured in a single replicate after 2-fold dilution, UC and EE samples were measured in triplo. Absorbance was read out using a Clariostar microplate reader at 750 nm or 561 nm, respectively, for both assays.

*Nanoparticle tracking analysis*

Scatter-based NTA was performed on a NanoSight NS500 instrument equipped with a 532 nm (green) continuous wave, < 80 mW laser and a CMOS High Sensitivity camera. The NanoSight NTA software, version 3.2 was used for analysis of the size distribution and number concentration of particles in the separated sEV fractions and blank controls, according to the manufacturer's guidelines. Latex transfer standards of 100 nm were freshly diluted, 1/1000 in Milli-Q ultrapure water, and measured at the start of each run as a quality control (Suppl. Materials Fig. S1). Samples obtained by the different methods SEC, EE and UC from one biological replicate were measured within the same run. Samples were diluted in PBS (SEC 1/20, EE 1/200 and UC 1/40) to ensure that the number of particles/frame was between 20 and 100, blanks were diluted in the same manner as the respective sample. Samples were loaded and measured using a syringe pump with infusion rate set at 5 for a constant flow rate. Each measurement consisted of 3 recordings of 60 seconds (1500 frames with 25 frames/second) using following settings: camera level 14-16, screen gain 1.0. Detection threshold 5 was used for analysis.

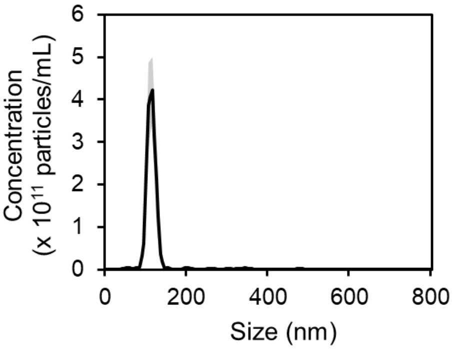


| Latex transfer standards | |
|---|---|
| Mean diameter (nm) | 120 ± 4 |
| Mode (nm) | 114 ± 3 |
| Particle concentration (x $10^{11}$ particles/mL) | 15 ± 1 |

**Supplementary Materials Figure M1.** Scatter-based NTA size distribution profile of 100 nm latex transfer standards. Data is represented as mean ± SEM of three independent dilutions (1/1000 in Milli-Q ultrapure water), each made on a different date and measured three times.

*Transmission electron microscopy*

A nickel TEM slot (Electron Microscopy Sciences, #FF2010-Ni) was placed on top of a droplet of the sEV sample (EE samples were 10 times diluted in PBS) or blank control on clean parafilm for 1 hour, before the slot with the absorbed fluid was washed three times with PBS and five times with ultrapure water for 2 minutes each. Ultrapure water was deprived of $CO_2$ by heating and all other solutions were passed through a 0.22 µm pore size filter before use. After a 10-minute fixation with 2.5% of

glutaraldehyde, the slot was again washed five times with ultrapure water for 2 minutes. Then, the slot was incubated in 2% uranyl acetate for 15 minutes, 0.13% methyl cellulose and 0.4% uranyl acetate for 10 minutes and dried at room temperature. Samples were examined using a Tecnai G2 Spirit BioTWIN TEM instrument in Bright Field modus at 120 kV. The output filament was about 5 µA. Images were taken at magnifications between 60,000 and 135,000 with a Veleta TEM CCD camera.

*Western blotting*

All products and equipment were bought from Bio-Rad Laboratories unless stated otherwise. Lysate of H1975 cells (10 ng/µL or 100 ng/µL of total protein concentration, depending on the analyzed protein marker) was used as a positive control and unprocessed unconditioned cell medium with 2% EV-depleted FBS (100 ng/µL) was used as a negative control. Equal volumes (36 µL) of the sEV samples, blank controls, positive and negative controls were mixed in a 3:1 ratio with 4× Laemmli Sample Buffer containing β-mercaptoethanol, and heated to 95°C for 5 minutes. Next, 40 µL of the prepared samples and 5 µL of Precision Plus Protein™ All Blue Prestained Protein Standards were loaded on a 4 - 20% Mini-PROTEAN® TGX Stain-Free™ Protein Gel. Proteins were separated by gel electrophoresis at 100 V using the PowerPac™ Basic Power Supply and transferred onto a 0.2 µm nitrocellulose membrane using the Trans-Blot Turbo RTA Mini 0.2 µm Nitrocellulose Transfer Kit (#1704270) and Trans-Blot Turbo Transfer System. Membranes were blocked for 2.5 hours at room temperature in 5% blotting grade blocker, consisting of non-fat dry milk in PBS containing 0.1% Tween-20 (PBS-T; Pierce 20X PBS Tween-20 Buffer). This was followed by overnight incubation while shaking at 4°C with primary antibodies for detection of CD81, Hsp70, CANX and rpS6 diluted 1/200 for detection of CD81 and rpS6 and 1/500 for detection of Hsp70 and CANX in solution 1 from the SignalBoost™ Immunoreaction Enhancer Kit (Millipore, Merck, #407207). The next day, the membranes were washed 6 times for 5 minutes with PBS-T prior to adding a horseradish peroxidase-conjugated donkey anti-mouse IgG (1/10,000, Jackson Immuno Research, #715-035-151) and incubated for 2 hours at room temperature in solution 2 from the SignalBoost™Immunoreaction Enhancer Kit. The membranes were again washed 6 times for 5 minutes with PBS-T and the signal was enhanced using the Clarity Western ECL Substrate (#1705061) according to the manufacturer's protocol before imaging the membranes with a Chemidoc XRS+ System. Processing of the images and densitometry analysis was performed using Image Lab Software, version 5.1.

*High-sensitivity flow cytometry: sample staining*

For high-sensitivity flow cytometry analysis, 180 µL of sEV samples obtained by SEC and UC and 400 µL of EE samples were fluorescently stained with 18 µL and 40 µL, respectively, of 10 µM of the dye Vybrant™ CFDA-SE (Thermo Fisher Scientific, #V12883) for 1 hour at room temperature in the dark. Next, 30 µL aliquots of the SEC and UC samples or 140 µL of EE samples were either left untreated (single-stained sample) or double-stained by adding 0.5 µg antibody to each sample: 10 µL of a PE-Cy7 fluorescently labeled mouse anti-human antibody against CD9 (50 µg/mL, BioLegend, San Diego, California, USA, #312115), 2.5 µL CD63 (200 µg/mL, BioLegend, #353009) or 2.5 µL CD81 (200 µg/mL, BioLegend, #349512), and incubated for another hour at room temperature in the dark. All antibodies were first centrifuged at 16,000 rcf for 5 min using a microcentrifuge 5415 R (Eppendorf) to avoid aggregates. After staining, samples were diluted to 300 µL with PBS, transferred to a 5 mL open-top thinwall polyallomer tube and purified by bottom-up density gradient centrifugation to remove the unbound dye. The density gradient was prepared by mixing 300 µL of stained sEV sample with 1 mL of 60% iodixanol (Optiprep, StemCELL Technologies, #07820). This was overlaid with 700 µL of 40% iodixanol in homogenization buffer containing 6 mM EDTA, 60 mM Tris-HCl and 0.25 mM sucrose (pH 7.4), 700 µL of 30% and finally 2 mL of 10% iodixanol in 1 mM EDTA, 10 mM Tris-HCl and 0.25 mM sucrose (pH 7.4). Bottom-up density gradient centrifugation was performed by overnight centrifugation at 366,613 rcf at 4°C using a SW55 Ti rotor in an Optima XPN-80 ultracentrifuge with moderate braking (acceleration 5/ deceleration 5, k-factor: 48). Next, 8 density gradient fractions of 480 µL each were collected from each sample starting from the top of the tube.

*High-sensitivity flow cytometry: measurement*

Prior to the sample measurements, PBS was measured as a negative control for which an event rate of < 10 events/second was accepted (Suppl. Fig. S15a). Yellow-Green fluorescent beads of 100 and 200 nm (Thermo Fisher Scientific, #F8803 and F8811) were diluted 10,000,000 times and then mixed in equal volumes. This mixture was used for setting gates to allow for small particle detection (Suppl. Materials Fig. S2). Small EVs were measured by fluorescence triggering on CFDA-SE with a set threshold of 0.30 in the FL-1 fluorescent channel (B530/40), and the gain set at 46.49 for FWSC, 50.42 for SSC, 55.06 for FITC and 70.00 for PE-Cy7. Sample fractions were diluted in a way that the event rate was below 10,000 events/second to avoid swarming effects, in case of SEC and UC 1/20 and for EE 1/100 in PBS. Gates were set on the FL-2 fluorescent channel (B750/LP) using CFDA-SE single-

stained samples, so that the percentage of positive events was ≈1%. Fractions were measured for 30 seconds with a sheath pressure of 4.9 and sample pressure of around 5.2, then the number of events was determined.

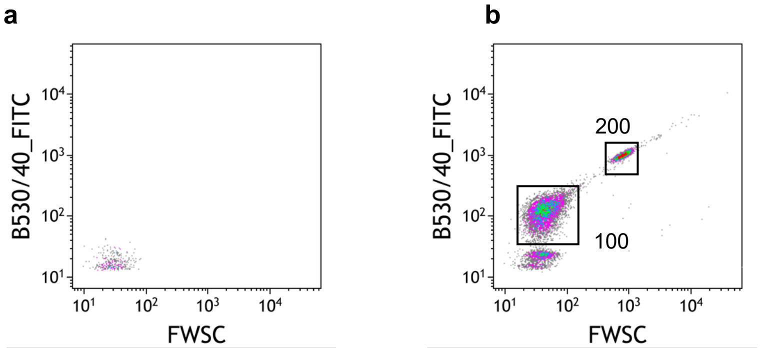


**Supplementary Materials Figure M2**. Flow cytometry analysis of (**a**) PBS as negative control and (**b**) 100 and 200 nm fluorescent polystyrene beads. Scatter plots or shown for forward scatter (FWSC) versus fluorescein isothiocyanate (FITC) fluorescence measured in the FL-1 fluorescent channel (B530/40). Yellow-Green fluorescent beads of 100 and 200 nm were diluted 10,000,000 times and then mixed in equal volumes. This mixture was used for setting gates to allow for small particle detection.

For each single-stained sample, the optiprep fraction containing most sEVs was determined. For all methods this was fraction 6 with a density of 1.10 g/mL as determined by Deville et al. (2021)[1], which is in accordance with studies that have previously described sEV densities between 1.10 and 1.19 g/mL[2]. This fraction was then used to prepare three replicate dilutions from each single- and double-stained sample, which were measured to obtain a mean percentage of sEVs staining positively for CD9, CD63 and/or CD81.

*High-sensitivity flow cytometry: fluorescence intensity calibration to units of mean equivalent soluble fluorochromes*

For the calibration of fluorescence intensities to units of MESF, PE-Cy7 MESF Beads (custom product manufactured by BD Biosciences) were measured using a BD Influx flow cytometer. Before measuring, 0.5 mL PBS supplemented with 0.5% BSA, pH 7.2 was added to get a concentration of 2 × $10^4$ of each population/tube. The beads were measured by triggering on FWSC with a threshold of 0.30 and with the gain set at 6.30 for FWSC, 13.10 for SSC and 70.00 for PE-Cy7. Here, it is important to note that the same settings for PE-Cy7 were used as for the sEV samples. A sheath pressure of 4.9 and sample pressure of 5.9 was used to measure 10,000 events at an event rate of 300.

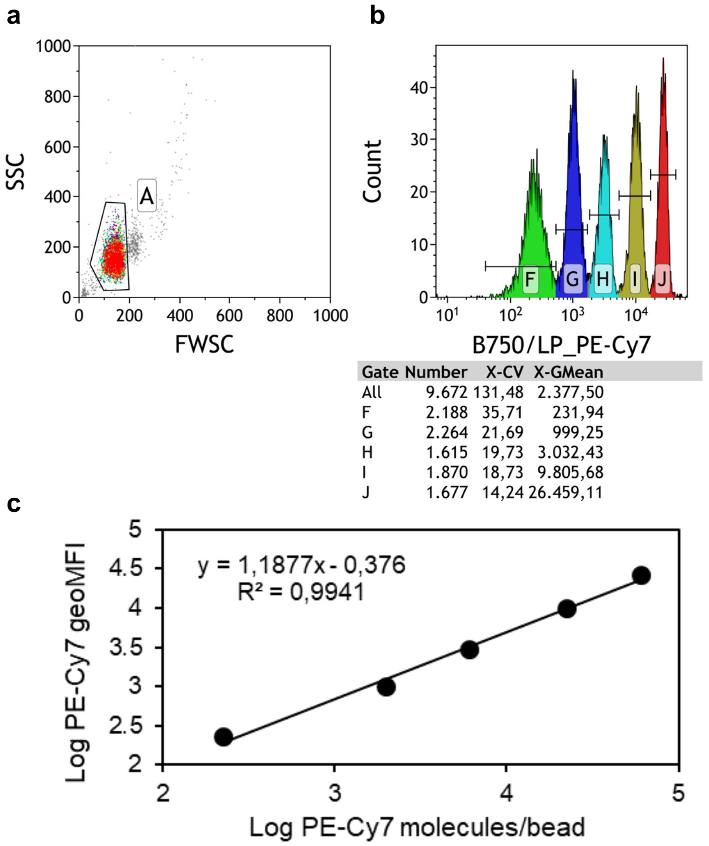


**Supplementary Materials Figure M3**. Flow cytometry of PE-Cy7 MESF Beads. (**a**) Side scatter (SSC, y-axis) is plotted against forward scatter (FWSC, x-axis). A gate 'A' was set around the positive population. (**b**) The number of positive events from population A defined by the gate (Count, y-axis) is plotted against the PE-Cy7 fluorescence intensity (B750/LP_PE-Cy7, x-axis). For each peak (F-J) the geoMFI (X-GMean) was calculated using the Kaluza Analysis Software, version 2.1. (**c**) Linear regression curve of log10 PE-Cy7 molecules/bead. The log10 of the geoMFI for each population of the PE-Cy7 MESF Beads was plotted against the log10 of the PE-Cy7 molecules/bead provided in the data sheet.

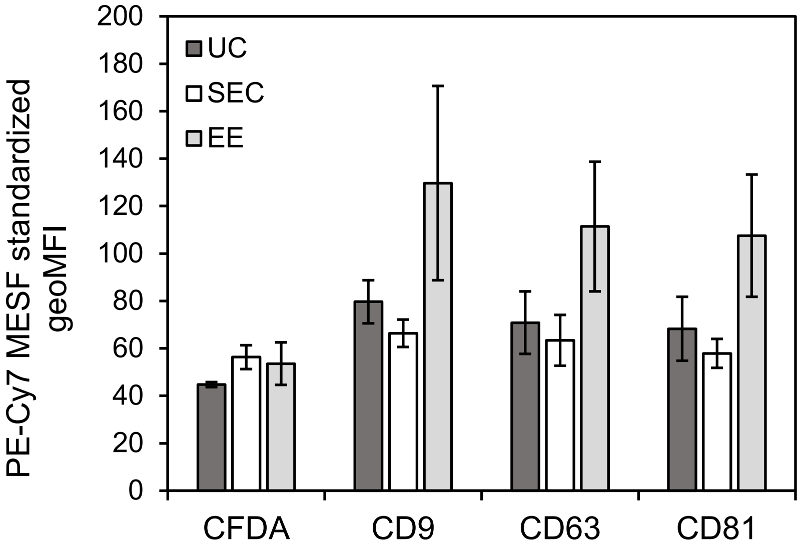


**Supplementary Materials Figure M4**. PE-Cy7 MESF standardized geometric mean fluorescence intensity (geoMFI) values obtained by flow cytometry analysis**.** PE-Cy7 fluorescence signals of CFDA-SE (CFDA) single stained, CFDA-SE/CD9 (CD9), CFDA-SE/CD63 (CD63) and CFDA-SE/CD81 (CD81) double stained sEV samples were measured in the FL-2 fluorescent channel. Values were converted using the above linear regression of Log10 PE-Cy7 molecules/bead (Suppl. Materials Fig. M3c). Data is represented as mean ± SEM (N = 2).

**Validation of RNase/DNase treatment**

The total extracted RNA and DNA content (both inside and outside the sEVs) was diluted to 500 µL and either left untreated or were treated with 5 µL RNase A/T1 (2 mg/mL RNase A; 5000 U/mL RNase T1), 1 µL DNase I (1 U/µL) and 50 µL 10× reaction buffer with MgCl2 for 30 minutes at room temperature, and thereafter DNase was inactivated by adding 25 µL of EDTA and heating the sample to 65°C for 10 minutes.

**RNase/DNase treatment of sEVs prior to RNA/DNA extraction**

For the EE workflow, this was carried out on-column after the initial washing step of the retained sEVs, by adding the nuclease mixture in a total of 500 µL Buffer XWP. After 1 minute of incubation at room temperature, the spin column was centrifuged for 5 minutes at 500 rcf in a swinging bucket Rotanta 460 R centrifuge. The solution that flowed through the spin column was added back on top and incubated for 25 minutes at room temperature before centrifugation for 1 minute at 500 rcf. A second washing step was carried out and the spin column was transferred to a fresh collection tube for elution. For the

SEC and UC workflows, the RNase/DNase mixture was added to the collected 500 µL sEV sample, incubated for 30 minutes at room temperature.

**Extraction of genomic DNA and cellular RNA with quality control from whole cells**

For preparation of cellular RNA or genomic DNA, which was used as cell control in Sanger sequencing (vide supra) or PCR reactions (vide infra), about 3 × $10^6$ cells grown in cell medium supplemented with 10% FBS were used and harvested as a cell pellet. Cells were lysed using 350 µL Buffer RLT Plus (Qiagen, #1053393) and homogenized with a QIAshredder (Qiagen, #79654). RNA was extracted using the RNeasy Plus Mini Kit (Qiagen, # 74134), eluted in 30 µL RNase-free water, and stored at -80°C until further use. The AllPrep DNA/RNA/miRNA Universal Kit (Qiagen, #80224) was used to isolate cellular genomic DNA per manufacturer's instructions.

Quality control of cellular RNA samples was performed with the Agilent RNA 6000 Nano assay (Agilent Technologies, #5067-1511) and the mRNA protocol on the Agilent Bioanalyzer 2100. RNA integrity numbers (RIN) were calculated with the Agilent 2100 Expert Software, mRNA Nano Series II to assess RNA degradation. Samples with RIN > 7.5 were accepted as intact RNA.

**Droplet digital PCR**

For ddPCR, cellular RNA samples were diluted to 10 ng/µL, sEV-derived nucleic acid samples after reverse transcription were used undiluted and sEV samples without RT treatment were 1:1 diluted with nuclease-free water. PPP-derived samples were used undiluted. Nuclease-free water was used as NTC. The master mix for each ddPCR-reaction contained 11 µL 2× ddPCR Supermix for Probes (No dUTP) (Bio-Rad Laboratories, #1863023), 1.1 µL 20× Primers and Probes, 7.4 µL or 3.9 µL nuclease-free water and 2.5 µL cellular-derived samples, 6 µL PPP-derived samples or the same volume of NTC. Droplets were generated using the QX200™ Droplet Generator per manufacturer's instructions and transferred to a 96-well plate before sealing the plate using the PX1 PCR Plate Sealer. PCR was performed in the C1000 Touch™ Thermal Cycler with 96–Deep Well Reaction Module using the following cycling conditions: 95°C for 10 minutes; 40 cycles at 94°C for 30 seconds, 55°C for 1 minute and 98°C for 10 minutes at a ramp rate of 2°C/second. Afterwards the plate was kept at 4°C for 30 minutes. Droplet fluorescence was read by the QX200™ Droplet Reader.
